# Use of whole blood in pediatric congenital heart surgery

**DOI:** 10.1016/j.xjon.2026.101925

**Published:** 2026-06-11

**Authors:** Laura Maitoza, Stephanie Bland, Precious Ann Fortes, Andrea McGonigle, Tiffany Williams, Glen Van Arsdell, Myke Federman

**Affiliations:** a Department of Pediatrics, Mattel Children's Hospital, University of California Los Angeles, Los Angeles, Calif; b Department of Cardiac Surgery, David Geffen School of Medicine, University of California Los Angeles, Los Angeles, Calif; c Wing-Kwai and Alice Lee-Tsing Chung Transfusion Service, Department of Pathology and Laboratory Medicine, David Geffen School of Medicine at UCLA, Los Angeles, Calif; d Department of Anesthesiology and Perioperative Medicine, David Geffen School of Medicine, University of California Los Angeles, Los Angeles, Calif

**Keywords:** cardiac surgery, cardiopulmonary bypass, congenital heart disease, whole blood

## Abstract

**Objectives:**

Cardiopulmonary bypass is necessary for surgical repair of complex congenital heart disease. We studied whether whole blood use in circuit prime and for initial intraoperative transfusion needs improved postoperative outcomes in infants less than 2 months of age.

**Methods:**

This was a single-center retrospective chart review of infants who underwent cardiac surgery using cardiopulmonary bypass between May 2019 and February 2023. We reviewed and analyzed intraoperative and postoperative variables for whole blood and standard blood prime use in patients, whereas the remainder of clinical management stayed unchanged.

**Results:**

Ninety-five patients were included in our analysis. Donor exposure was reduced in the whole blood group with a median of 3 donors (Q1-Q3: 2-4) compared with the standard group with 5 donors (Q1-Q3: 5-6, *P* < .001). The whole blood cohort used whole blood for intraoperative transfusion needs over individual blood components. Whole blood recipients had lower postoperative platelet counts and fibrinogen levels, but no clinical markers of increased postoperative bleeding compared with the standard group. There was no statistically significant difference in length of stay, mechanical ventilation days, postoperative transfusion requirements, or time to delayed sternal closure between groups.

**Conclusions:**

The whole blood cohort was found to have fewer donor exposures and less intraoperative individual blood component use but no difference in postoperative outcomes. Continued research must be pursued to understand if whole blood with new circuit techniques and hemostatic interventions can reduce transfusion needs and lead to superior results.

**Figure:**
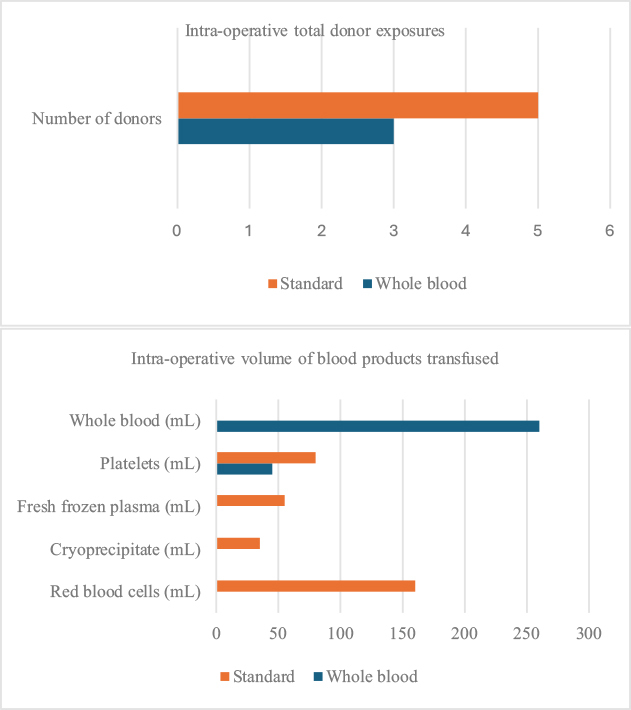
Median intraoperative blood volumes and donor numbers for WB and standard groups.

**Central Message**

WB prime for congenital cardiac surgery is resource intense but demonstrates a reduction in donor exposures and intraoperative blood component use compared with standard blood prime.

**Perspective**

This was a single-center study to investigate an intervention using WB in circuit prime and for intraoperative transfusion needs to potentially reduce postoperative bleeding, edema, and blood product exposure for infants undergoing CPB for congenital heart repairs with the goal to improve postoperative outcomes by influencing the postbypass inflammatory response.

Congenital heart disease (CHD) is the most common birth defect affecting 1 to 2% of all live births. Of those born with CHD, approximately 30% are born with complex CHD, requiring a cardiac surgical intervention, including cardiopulmonary bypass (CPB), within the first year of life.^1^ With more children surviving to adulthood, the need for additional interventions, including orthotopic heart transplantation, increases. Given this potential, the field must consider the blood transfusion needs required over the course of a lifetime with the potential for multiple heart operations.^2^, ^3^, ^4^, ^5^, ^6^ Increased donor exposures can affect future transplant risk and contribute to postoperative morbidity and mortality due to the increased chance for transfusion-related reactions.^2^^,^^3^^,^^7^^,^^8^

Other studies have investigated ways to reduce blood product exposure given the concerns for increased inflammation and myocardial dysfunction due to blood products in the perioperative period.^4^^,^^9^ Some ways to reduce transfusion needs include antifibrinolytic infusions, autologous blood collection pre-CPB, and the use of recombinant factors post-CPB.^10^, ^11^, ^12^, ^13^, ^14^, ^15^, ^16^, ^17^ Early studies comparing whole blood (WB) prime with standard prime failed to show any benefit and in fact demonstrated that circuit priming with WB was associated with increased length of stay (LOS) in the cardiac intensive care unit (CICU).^9^ In the 20 years since this article was published, the management of infants with CHD has evolved, so the use of WB may have a more beneficial impact in the current era.

One significant prior study using reconstituted WB prime for neonates did demonstrate a reduction in mechanical ventilation (MV) days and postoperative LOS.^18^ Finding ways to hasten postoperative recovery, reduce morbidity, and improve neurodevelopmental outcomes are essential as new and improved surgical and perfusion techniques arise. Miniaturized bypass circuits and reduced circuit prime volume may reduce the inflammatory effect of CPB. Postoperative care efforts are aimed at early extubation and mobility, reduced neurosedative use, and protocolized nutrition advancement.

Our multidisciplinary group developed a WB program for infants undergoing cardiac surgery that was officially instituted in May 2019. We chose infants who were 2 months of age or younger for this initiative because we thought that they would be most likely to benefit from this resource intensive effort, which took approximately 6 months of preliminary planning and work before the first use of WB in the operating room (OR). The aim of our analysis was to see if using a WB prime and WB for initial transfusion needs in the OR would decrease intraoperative transfusion requirements for infants undergoing complex congenital heart surgery along with improving secondary outcomes of postoperative bleeding, hospital LOS, duration of MV, and time to chest closure.

## Materials and Methods

After Institutional Review Board (IRB) approval (IRB #23-000651, May 15, 2023, IRB exempt), we carried out a retrospective chart review of term infants less than 2 months of age who underwent cardiac surgery using CPB from May 2019 to February 2023. We excluded 8 infants (5 WB, 3 standard) who required extracorporeal membrane oxygenation support in the first 48 hours post-CPB. Perfusion and anesthesia records were reviewed along with intensive care data from the electronic medical record. Two infants in the WB group were older (63 and 64 days of age) due to last minute surgical schedule changes.

All intraoperative and postoperative care for both groups were at the discretion of the surgeons, anesthesiologists, perfusionists, and intensivists. Per protocol, all infants undergoing cardiac surgery had a nasal or oral endotracheal tube with peripheral arterial and central venous access. A dextrose-containing solution is used as the carrier for maintenance fluids. Vasoactive drugs used intraoperatively and postoperatively include epinephrine, milrinone, vasopressin, and nitroprusside. All patients are maintained on antifibrinolytic infusions throughout the intraoperative phase, which are initiated after central venous access is obtained.

Collection of WB occurred within 5 days of the surgical date. When patients are scheduled for surgery, the surgical team provides the blood bank (BB) and donor recruitment services with the name, medical record number, and date of surgery via e-mail. A BB supervisor confirms the patient's blood type and the absence of maternal antibodies. If maternal antibody is present that is incompatible with type specific WB or if the surgical timing is an emergency, those patients were ineligible for WB. Donor recruitment reviewed and screened eligible donors, placing a note on the electronic donors' files with the WB recipient's name, medical record number, and date of birth, indicating “Pediatric Cardiac Surgery WB.” Once the phlebotomist completed WB collection, they place an orange tie tag on the donor unit. When the component processing laboratory receives the donation, the tie tag alerts to keep the unit as WB for the assigned patient.

For donor exposures, platelets and plasma are not pooled from multiple donors at our institution. Cryoprecipitate in our institution is available as single units or unit pools, so donor exposure was calculated based on transfusion event by unit. The number of donor exposures was calculated for packed red blood cells (pRBCs), platelets, and plasma. Multiple transfusions could come from 1 donor if dosed as aliquots. Each transfusion event for WB corresponded to a single WB unit and therefore 1 donor exposure.

The BB provides 2 units of WB for the OR; one 500-mL unit and a second unit split into two 250-mL aliquots. Priming of the CPB circuit is performed by first adding 50 mL of 25% albumin to coat the circuit tubing, followed by 300 mL of WB transfused into the cardiotomy. Prime drugs are added, including 2000 units heparin, 10 mEq sodium bicarbonate, 75 mg calcium chloride, and 40 mg tranexamic acid. The dosages of sodium bicarbonate and calcium are reduced to half of typical dosages because WB is less acidotic than washed pRBC conventionally used to prime the bypass circuit. During the rewarming phase, the remaining 200 mL of WB is added sporadically to the bypass circuit to optimize the patient from a hematologic standpoint for weaning CPB.

For standard blood prime, the CPB circuit is primed with 1 unit (250-300 mL) of washed pRBC and 100 mL fresh-frozen plasma (FFP). The remaining volume of FFP (100-150 mL) is given into the circuit before termination of bypass. The prime drugs remain the same except for the sodium bicarbonate, which is 20 mEq for standard protocol.

Standard laboratories during rewarming before separation from bypass include platelet count, fibrinogen level, and rotational thromboelastometry. The hematocrit in a WB unit is typically 30 to 40%, lower than a pRBC unit, requiring ultrafiltration on bypass to maintain the patient's hematocrit more than 30%. Target hematocrits for patients depend on their underlying anatomy with goal hematocrits more than 30% for cyanotic and 24 to 30% for acyanotic lesions. After CPB is terminated, modified ultrafiltration is performed using the conventional arteriovenous system for all patients with the addition of more WB or blood components, depending on the patient's volume status, hemodynamics, and blood availability. Transfusions are performed to obtain platelet counts greater than 150 × 10^3^/μL, fibrinogen greater than 100 mg/dL and international normalized ratio less than 1.5. A discussion between the surgeon and the anesthesiologist occurs to assess the surgical field for ongoing bleeding in combination with laboratory-based evidence of coagulopathy.

For statistical analysis, continuous data are described as medians with interquartile ranges and means with SDs as appropriate. Any missing values on a given variable were excluded from the analysis. In addition to treatment group comparisons, we divided the blood groups into the Society of Thoracic Surgeons-European Association for Cardio-Thoracic Surgery (STAT) subgroups for further analysis. The *P* values for comparing continuous variables between blood treatment groups were computed using the nonparametric Wilcoxon rank-sum test because most outcomes did not follow a normal distribution on the original scale. The *P* values for comparing categorical variables were computed using the Fisher exact test.

We investigated the effect of an infant receiving WB on 4 separate outcomes: (1) The sternum was left open or closed, (2) the time on MV in days, (3) the LOS in days, and (4) the time to delayed sternal closure (DSC) in days. Adjustment for 3 covariates, STAT, cyanotic status, and preoperative weight, was carried out using inverse propensity weighting (IPW) where the propensity weights were computed using a binary logistic regression of WB treatment on the covariates. MV and LOS in days were found to have a normal distribution on the log base 10 scale. For these 2 outcomes, the IPW adjusted log scale means were first computed and the base 10 antilog of these means were computed to give the original scale adjusted medians. IPW adjusted proportions of the percent with an open or closed sternum were computed using IPW weighted logistic regression. The median days to DSC was computed using an IPW weighted quasi-Poisson regression because the distribution of days to DSC had an approximate Poisson distribution. All analyses were performed in R 4.3.2 (R project for statistical computing).

## Results

Of the 95 infants included in this review, 45 received standard blood prime and 50 received WB prime. The study groups were concurrent. Three infants were difficult to crossmatch due to antibodies, and the remainder of those in the standard group had abrupt changes in surgical dates or emergency scheduled surgeries that did not allow the necessary time for WB to be assigned to their case. Baseline characteristics were similar between the 2 groups in reference to age at surgery, birth weight, STAT category, preoperative creatinine and hemoglobin levels, and CPB and crossclamp times (Tables 1 and 2). There were no deaths within the 2 groups before discharge from the hospital.

**Table 1:** Baseline demographics shown as median distribution with interquartile range

Patient characteristics	Whole blood (n = 50)	Standard blood (n = 45)	*P* value
	Median (IQR)	Median (IQR)	
Age (d)	6 (4.0-9.0)	6 (3.0-19.0)	.37
Birth weight (kg)	3.36 (2.9-3.6)	3.3 (2.9-3.7)	.95
Crossclamp (min)	76 (66.0-103.0)	71 (58.0-110.0)	.38
CPB (min)	120 (101.3-149.8)	124 (101.0-142.0)	.80
Preoperative Hgb g/dL	12.8 (11.9-14.6)	13.2 (12.3-14.5)	.73
Preoperative creatinine, mg/dL	0.42 (0.30-0.53)	0.46 (0.35-0.56)	.45
Postoperative ICU Hgb	12.7 (10.6-14.7)	12.1 (10.5-13.7)	.47
Postoperative ICU Plt, ×10^3^/μL	143.5 (99.8-182)	174 (140-241)	.005
Postoperative ICU Fib, mg/dL	194 (171.3-238.5)	254.5 (230-291.8)	<.001
Vasoactive inotrope score	15.5 (13-20.4)	17 (12-20.7)	.63

*IQR*, Interquartile range; *CPB*, cardiopulmonary bypass; *Hgb*, hemoglobin; *ICU*, intensive care unit; *Plt*, platelet; *Fib*, fibrinogen.

**Table 2:** Baseline demographics shown as mean distribution with SD

Patient characteristics	Whole blood (n = 50)	Min	Max	Standard blood (n = 45)	Min	Max
	Mean (SD)			Mean (SD)		
Age (d)	12.18 (15.54)	2	64	14.69 (18.14)	1	68
Birth weight (kg)	3.28 (0.51)	1.8	4.3	3.30 (0.59)	2	4.8
Crossclamp (min)	90.94 (38.99)	34	206	89.29 (43.48)	46	247
CPB (min)	135.74 (58.36)	41	403	129.93 (50.82)	57	341
Preoperative Hgb, g/dL	13.126 (2.18)	8.1	18.5	13.05 (2.17)	7.3	17.2
Preoperative creatinine, mg/dL	0.4294 (0.169)	0.12	1.07	0.45 (0.167)	0.18	0.92
Postoperative ICU Hgb	12.8 (2.65)	7.5	18.6	12.5 (2.32)	7.7	19
Postoperative ICU Plt, ×10^3^/μL	151.1 (72.9)	59	393	194.5 (82.5)	43	400
Postoperative ICU Fib, mg/dL	210.3 (67.5)	41	450	266.6 (55.7)	183	390
Vasoactive inotrope score	16.1 (5.3)	6	25.7	16.9 (6.76)	3	42

*CPB*, Cardiopulmonary bypass; *Hgb*, hemoglobin; *ICU*, intensive care unit; *Plt*, platelet; *Fib*, fibrinogen.

### Laboratory Findings

There was no significant difference in first intraoperative hemoglobin level or platelet count obtained in the OR. Postoperative laboratory results were sent within the first hour of return to the CICU. The median postoperative fibrinogen level was lower in the WB group at 194 mg/dL (Q1-Q3: 171.3-238.5) compared with the standard group at 254.5 mg/dL (Q1-Q3: 230-291.8, *P* < .001). The median postoperative platelet count was lower in the WB group at 143.5 × 10^3^/μL (Q1-Q3: 99.8-182) compared with the standard group at 174 × 10^3^/μL (Q1-Q3: 140-241, *P* = .005). There was no statistically significant difference between postoperative hemoglobin level or international normalized ratio or maximum postoperative creatinine level within the first 48 hours (Tables 1 and 2).

### Transfusions

The WB group had a statistically significant lower median transfusion volume of all intraoperative blood components (pRBCs, FFP, platelets, cryoprecipitate) compared with the standard group (Tables 3 and 4). The median number of donor exposures from blood products transfused in the OR was 3 donors (Q1-Q3: 2-4) for WB compared with 5 donors (Q1-Q3: 5-6) for standard, which is statistically significant (*P* < .001, Tables 3 and 4). There was no statistically significant difference in postoperative transfusion requirements within the first 24 hours of arrival to CICU when comparing the 2 groups (Tables 5 and 6). Table E1 shows the number of patients receiving components for each group.

**Table 3:** Operating room transfusion volumes and donor numbers shown as median distribution with interquartile range

OR outcome measures	Whole blood (n = 50)	Standard blood (n = 45)	*P* value
	Median (IQR)	Median (IQR)	
Packed red blood cells	0 (0-117.5)	160 (110-275)	<.001
Cryoprecipitate	0 (0-23.8)	35 (23-46)	<.001
Fresh-frozen plasma	0 (0-0)	55 (40-100)	<.001
Platelets	45 (0-55)	80 (55-110)	<.001
Whole blood	260 (168.8-419.4)	0 (0-0)	<.001
Cell Saver	0 (0-0)	0 (0-40)	.33
No. of donors	3 (2-4)	5 (5-6)	<.001

Volume in milliliters.

**Table 4:** Operating room transfusion volumes and donor numbers shown as mean distribution with SD

OR outcome measures	Whole blood (n = 50)	Min	Max	Standard blood (n = 45)	Min	Max
	Mean (SD)			Mean (SD)		
Packed red blood cells	59.44 (92.1)	0	317	199.1 (155.9)	0	606
Cryoprecipitate	12.82 (20)	0	80	35.6 (18.5)	0	94
Fresh-frozen plasma	7.4 (35.8)	0	245	71.6 (51.6)	0	210
Platelets	44.8 (45.1)	0	170	91.2 (45.1)	35	281
Whole blood	314.37 (171.3)	120	850	0.0 (0)	0	0
Cell saver	26.4 (59.3)	0	300	24.7 (39.9)	0	155
No. of donors	3.26 (2.01)	1	11	5.51 (1.95)	2	11

Volume in milliliters.

**Table 5:** Cardiac intensive care unit blood transfusion volumes and chest tube output shown as median distribution with interquartile range

CICU outcome measures	Whole blood (n = 50)	Standard blood (n = 45)	*P* value
	Median (IQR)	Median (IQR)	
Packed red blood cells	0 (0-35)	0 (0-35)	.53
Cryoprecipitate	0 (0-0)	0 (0-0)	.18
Fresh-frozen plasma	0 (0-0)	0 (0-0)	.92
Platelets	0 (0-0)	0 (0-0)	.88
Chest tube output, 0-24 h	96.5 (69.2-124)	102 (80-140)	.21
Chest tube output, 24-48 h	35.4 (25-61.5)	49 (32-66.5)	.17

Volume in milliliters.

**Table 6:** Cardiac intensive care unit blood transfusion volumes and chest tube output shown as mean distribution with SD

CICU outcome measures	Whole blood (n = 50)	Min	Max	Standard blood (n = 45)	Min	Max
	Mean (SD)			Mean (SD)		
Packed red blood cells	17 (27.9)	0	112	19.9 (28.8)	0	120
Cryoprecipitate	0.8 (4.1)	0	22	0 (0)	0	0
Fresh-frozen plasma	2.2 (8.9)	0	45	2.2 (8.6)	0	45
Platelets	3 (10.4)	0	45	4.3 (15.8)	0	90
Chest tube output, 0-24 h	97.1 (38.5)	19.1	191	110.2 (46.5)	40	234
Chest tube output, 24-48 h	45.1 (27.2)	4.5	113	50.5 (23.3)	10	107.5

Volume in milliliters.

### Postoperative Management

There was no significant difference between groups in maximum postoperative vasoactive inotrope score (Tables 1 and 2) or chest tube output within the first 48 hours (Tables 5 and 6). The difference in number of WB compared with the standard patients who underwent DSC was not statistically significant (*P* = .058). However, when adjusting for STAT category, preoperative weight, and presence of cyanotic heart disease, there was a statistically significant difference in the use of DSC between groups (*P* = .02, Table 7). For all 95 patients, the median time to sternal closure was not significantly different between groups (Table 8).

**Table 7:** Medians for chest closed percentages between groups (unadjusted and adjusted for preoperative birth weight, Society of Thoracic Surgeons-European Association for Cardio-Thoracic Surgery category, and postoperative cyanosis)

Treatment	n	Unadjusted	SE	*P* value	Adjusted	SE	*P* value
		Median			Median		
Standard	45	26.7%	6.6%	.06	29.2%	4.7%	.02
Whole blood	50	46.0%	7.0%		45.4%	5.1%

**Table 8:** Medians for days to chest closure between groups (unadjusted and adjusted for preoperative birth weight, Society of Thoracic Surgeons-European Association for Cardio-Thoracic Surgery category, and postoperative cyanosis)

Treatment	n	Unadjusted	SE	*P* value	Adjusted	SE	*P* value
		Median			Median		
Standard	45	4.0	0.62	.10	3.8	0.51	.35
Whole blood	50	2.5	0.57		3.2	0.46	

**Figure E1:**
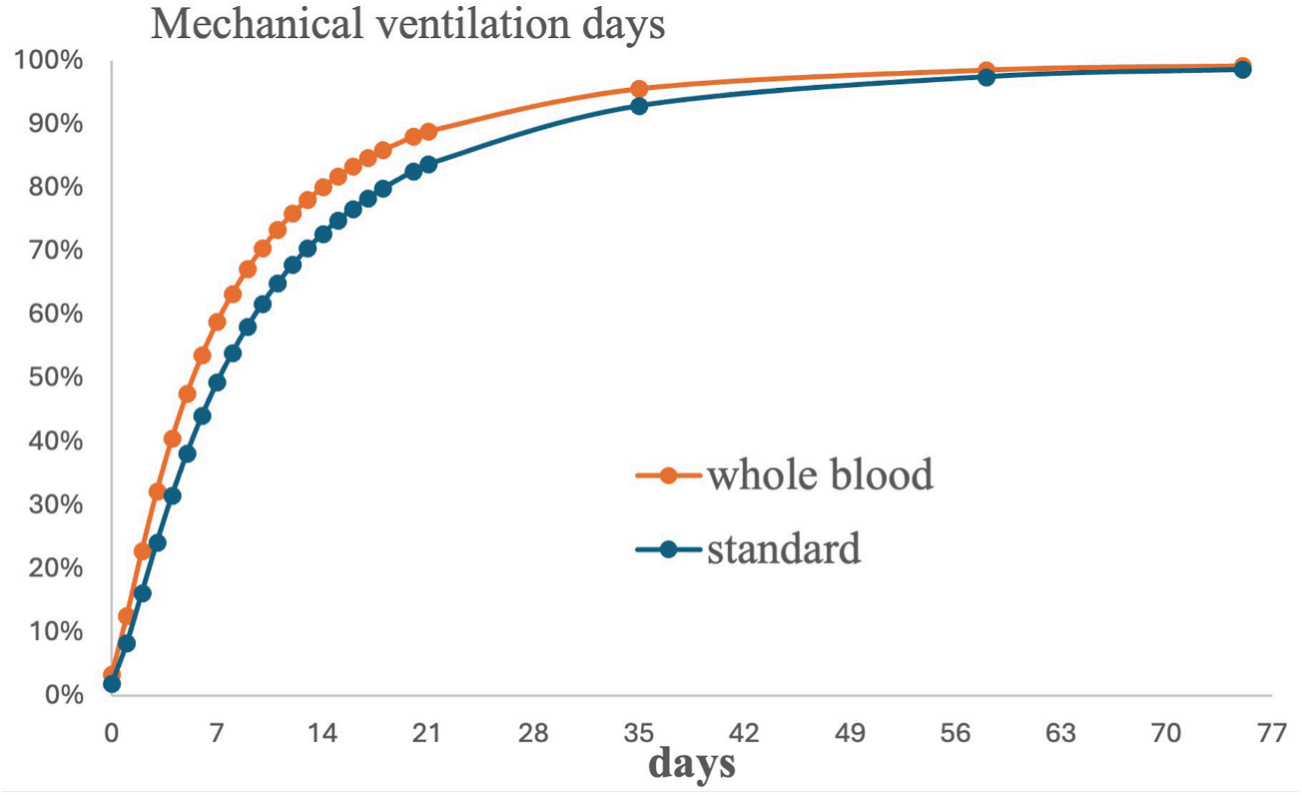
Covariate adjusted cumulative distribution for MV days.

**Figure E2:**
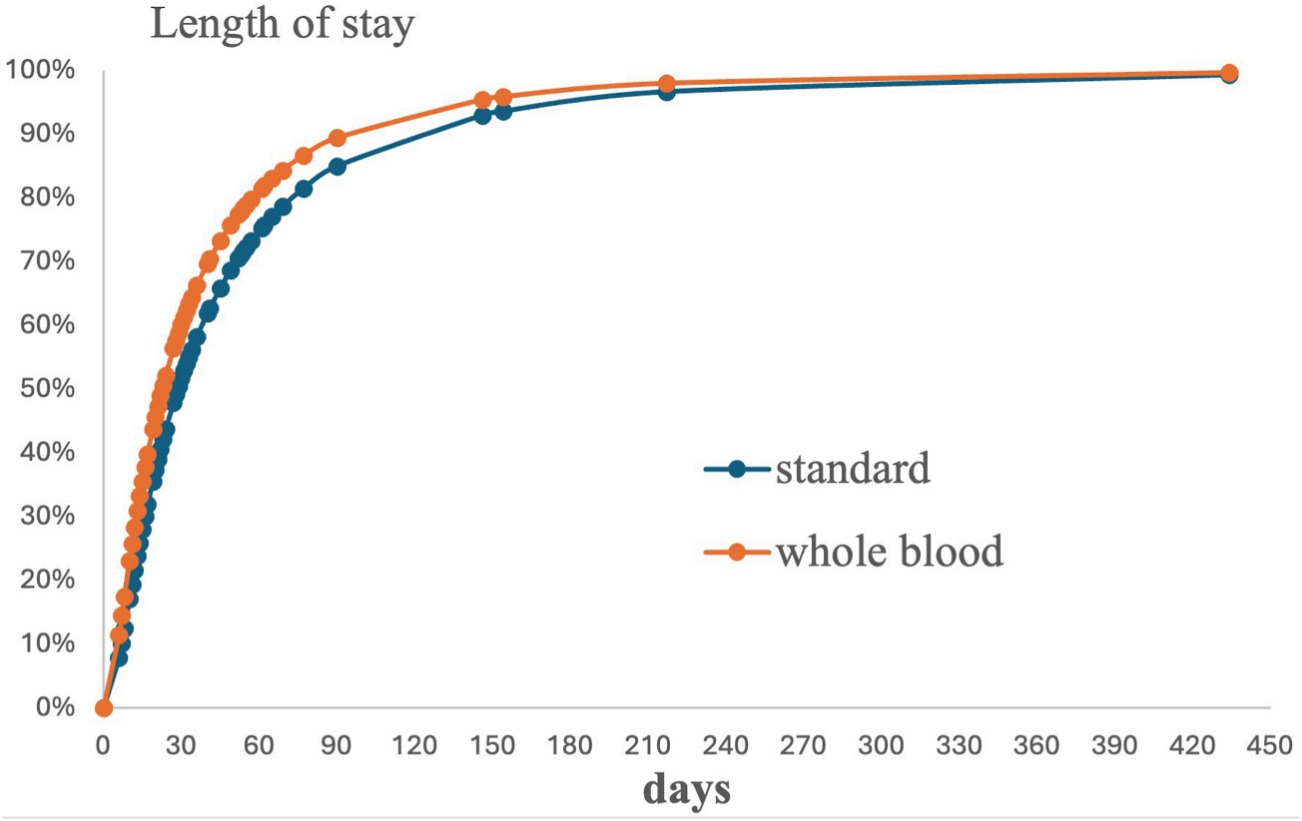
Covariate adjusted cumulative distribution for LOS.

The median number of days of postoperative MV was lower in the WB group (5 days [SE, 1.14]) compared with the standard group (7 days [SE, 2.42], *P* = .045). When controlling for STAT category, preoperative weight, and presence of cyanotic heart disease, this difference did not remain significant (Table 9 and Figure E1). The WB group (19 days [SE, 12.19]) had a shorter median LOS compared with the standard group (28 days [SE, 6.11], *P* = .049). When controlling for STAT category, preoperative weight, and presence of cyanotic heart disease, this difference was not statistically significant (Table 10 and Figure E2).

**Table 9:** Medians for mechanical ventilation days between groups (unadjusted and adjusted for preoperative birth weight, Society of Thoracic Surgeons-European Association for Cardio-Thoracic Surgery category, and postoperative cyanosis)

Treatment	n	Unadjusted	SE	*P* value	Adjusted	SE	*P* value
		Median			Median		
Standard	45	7.0	2.42	.04	7.1	0.84	.10
Whole blood	49	5.0	1.41		5.4	0.67

**Table 10:** Medians for postoperative length of stay between groups (unadjusted and adjusted for preoperative birth weight, Society of Thoracic Surgeons-European Association for Cardio-Thoracic Surgery category, and postoperative cyanosis)

Treatment	n	Unadjusted	SE	*P* value	Adjusted	SE	*P* value
		Median			Median		
Standard	43	28.0	6.11	.05	28.6	3.34	.16
Whole blood	47	19.0	12.19		22.6	2.65	

### Society of Thoracic Surgeons-European Association for Cardio-Thoracic Surgery Categories

The same analyses were repeated within groups of STAT categories. Key comparisons are outlined in Table E2, Table E3, Table E4, Table E5.

## Discussion

The development of a WB program is complicated and resource intensive. Developing the capability to provide WB for an infant during cardiac surgery required a multidisciplinary effort among transfusion medicine, the blood donor center, cardiac surgery, cardiac anesthesia, perfusion services, and the preoperative clinical team. After several months of preparation, we were able to develop a process to identify surgical cases for which WB was necessary and provide WB to the OR team. Rapid communication among key stakeholders is required to meet our predefined requirement that WB be given within 5 days of collection. We are fortunate to have an on-site blood collection and donation center, which identifies multiple potential donors for each case to ensure successful collection and processing of 1 to 2 units of WB for each patient to be reserved and issued on the date of surgery.

This review of single-center data from 2019 to 2023 demonstrates some key outcome differences in infants who receive WB compared with standard blood during CPB operations for CHD. In this study, we identified decreased donor exposure among patients receiving WB prime compared with standard blood. Evidence of fewer donor exposures has been reported in prior studies evaluating the use of WB in cardiac surgery.^2^^,^^3^^,^^7^^,^^8^ A single-center study also found fewer donor exposures in using WB versus components for liver transplant recipients.^19^ Reducing donor exposures during cardiac surgery has many potential benefits, including decreased risk for transfusion reactions, transfusion-related acute lung injury, transfusion-associated circulatory overload, and infectious disease transmission. Children with complex CHD may eventually require heart transplantation, and human leukocyte antigen sensitization from blood product exposure creates significant challenges in finding a suitable organ.

Patients who received WB required fewer days of postoperative MV and had a shorter postoperative LOS compared with patients receiving standard blood, although there were no statistically significant outcomes when adjusting for selected covariates. Potential reasons why WB recipients could be extubated sooner include signs in the OR of reduced inflammation, leading to chest closure, which accelerates postoperative recovery. A recent study found no change in multiple organ dysfunction (new occurrence or progression) with fresh versus standard storage pRBC units.^20^

Postoperatively, we did not find differences in transfusion requirements in the first 24 hours or in volume of chest tube drainage, used as a surrogate for bleeding, in the first 24 hours. Early fibrinogen level and platelet counts were lower in the WB group, although this was not associated with more bleeding or increased transfusion volume. Prior studies have shown that fresh WB is associated with improved hemostasis and platelet function post-CPB,^2^^,^^8^^,^^9^^,^^21^, ^22^, ^23^, ^24^, ^25^, ^26^, ^27^ but we were not able to demonstrate this finding in our cohort. Known risk factors that affect postoperative bleeding include patient age and surgical complexity, which is why we studied infants and performed a subgroup analysis by STAT category.^28^ In the STAT 5 subgroup analysis, we did find a reduction in postoperative chest tube drainage in the WB group.

### Limitations

The limitations to this study include that it is a retrospective single-center study. The staff in the OR were not blinded, and the patients were not randomized. Patient care in the OR and CICU was determined by the care team, including the decision to transfuse. The number of WB units sent to the OR per patient increased from 1 to 2 units during the study, allowing for additional transfusion beyond the circuit prime. The age of WB used was 3 to 5 days old, but we did not distinguish between WB of different ages. We did not look at the age of the blood products used in the standard blood group. The sample size is not adequate, particularly with covariate adjustment, and a larger study population is needed to better understand potential outcome advantages for the WB group, including LOS and MV days.

## Conclusions

In this retrospective analysis, we demonstrate that the use of WB for CPB circuit priming and transfusion in the OR in pediatric cardiac surgery reduces donor exposure, intraoperative blood component transfusions, and need for DSC. Further research is necessary to understand the effects of WB on postoperative outcomes given no differences seen in our study population. Other methods that may replicate the potential benefit of WB should be further studied, including the use of reconstituted fresh WB, application of conservative intraoperative and postoperative transfusion goals, reduction in the age of transfused blood products, and implementation of perfusion and hemostatic techniques to reduce the need for blood transfusions and to combat postoperative coagulopathy.

## Conflict of Interest Statement

The authors reported no conflicts of interest.

The *Journal* policy requires editors and reviewers to disclose conflicts of interest and to decline handling or reviewing manuscripts for which they may have a conflict of interest. The editors and reviewers of this article have no conflicts of interest.

## Figures and Tables

**Table E1:** Number of whole blood and standard blood patients receiving component transfusions in the operating room and cardiac intensive care unit

Blood products	Whole blood	Standard blood
OR cell saver	11	16
OR packed red blood cells	20	38
OR cryoprecipitate	18	43
OR fresh-frozen plasma	4	43
OR platelets	32	45
CICU packed red blood cells	17	19
CICU cryoprecipitate	2	0
CICU fresh-frozen plasma	3	3
CICU platelets	4	4

*OR*, Operating room; *CICU*, cardiac intensive care unit.

**Table E2:** Unadjusted outcome variable results for whole blood and standard prime blood groups by Society of Thoracic Surgeons-European Association for Cardio-Thoracic Surgery 1-3 subgroup

STAT 1-3 outcome measures	Whole blood (n = 16)	Standard blood (n = 8)	*P* value
	Median (IQR)	Median (IQR)	
No. of OR donors	2.5 (1.8-3.0)	4 (3-5)	.05
OR pRBC, mL	0 (0-91.3)	131 (0-162.3)	.05
OR cryoprecipitate, mL	0 (0-4.7)	24.0 (21.5-36.2)	.005
OR FFP, mL	0 (0-0)	55.0 (40.0-128.7)	<.001
OR platelets, mL	40 (0-55)	55 (55-87.5)	.04
Postoperative ICU Hgb, g/dL	13 (11.2-13.8)	10.2 (9.8-12)	.04
Postoperative ICU Plt, ×10^3^/μL	146 (80-175.3)	236 (156.5-257.3)	.05
Postoperative ICU Fib, mg/dL	221.5 (170.8-250.3)	246.5 (221.5-268.5)	.22
Postoperative ICU INR	1.4 (1.3-1.43)	1.4 (1.38-1.5)	.49
Sternal closure time, d	0 (0-3)	1 (0-4)	.74
Chest tube output, 0-24 h, mL	82.5 (54.7-105.7)	73.5 (54.9-121.3)	1.00
Chest tube output, 24-48 h, mL	35.7 (27.1-52.0)	42.5 (27.6-59.2)	.65

Volumes in milliliters. *IQR*, Interquartile range; *OR*, operating room; *pRBC*, packed red blood cells; *FFP*, fresh-frozen plasma; *ICU*, intensive care unit; *Hgb*, hemoglobin; *Plt*, platelet; *Fib*, fibrinogen; *INR*, international normalized ratio.

**Table E3:** Unadjusted outcome variable results for whole blood and standard prime blood groups by Society of Thoracic Surgeons-European Association for Cardio-Thoracic Surgery 4-5 subgroup

STAT 4-5 outcome measures	Whole blood (n = 34)	Standard blood (n = 37)	*P* value
	Median (IQR)	Median (IQR)	
No. of OR donors	3 (2-4)	5 (5-7)	<.001
OR pRBC, mL	0 (0-125)	185 (115-280)	<.001
OR cryoprecipitate, mL	0 (0-28)	38 (23-46)	<.001
OR FFP, mL	0 (0-0)	55 (35-80)	<.001
OR platelets, mL	52.5 (0-66.3)	85 (60-110)	<.001
Postoperative ICU Hgb, g/dL	12.6 (10.6-15.1)	12.9 (11.1-14.4)	.87
Postoperative ICU Plt, ×10^3^/μL	138 (108.5-182)	172 (137-227)	.05
Postoperative ICU Fib, mg/dL	190.5 (172-219.3)	256 (234.8-298.8)	<.001
Postoperative ICU INR	1.4 (1.3-1.5)	1.5 (1.3-1.6)	.37
Sternal closure time, d	3 (0-6)	4 (2-5)	.24
Chest tube output, 0-24 h, mL	99.1 (70.3-131.9)	104.6 (82-140)	.31
Chest tube output, 24-48 h, mL	33.5 (24.8-65.7)	54 (34-66.5)	.31

Volumes in milliliters. *IQR*, Interquartile range; *OR*, operating room; *pRBC*, packed red blood cells; *FFP*, fresh-frozen plasma; *ICU*, intensive care unit; *Hgb*, hemoglobin; *Plt*, platelet; *Fib*, fibrinogen; *INR*, international normalized ratio.

**Table E4:** Unadjusted outcome variable results for whole blood and standard prime blood groups by Society of Thoracic Surgeons-European Association for Cardio-Thoracic Surgery 4 subgroup

STAT 4 outcome measures	Whole blood (n = 21)	Standard blood (n = 24)	*P* value
	Median (IQR)	Median (IQR)	
No. of OR donors	3 (2-3)	5 (5-6)	<.001
OR pRBC, mL	0 (0-20)	140.0 (75.0-253.8)	<.001
OR cryoprecipitate, mL	0 (0-25)	38 (23-46)	<.001
OR FFP, mL	0 (0-0)	55.0 (35.0-62.5)	<.001
OR platelets, mL	40 (0-55)	80 (58.8-110)	<.001
Postoperative ICU Hgb, g/dL	12.2 (10.5-14.1)	11.8 (10.5-13.2)	.87
Postoperative ICU Plt, ×10^3^/μL	128 (99-149)	171 (132-223.5)	.03
Postoperative ICU Fib, mg/dL	183 (170-212)	251 (228.5-270.5)	<.001
Postoperative ICU INR	1.4 (1.4-1.6)	1.5 (1.4-1.6)	.33
Sternal closure time, d	3 (0-4)	4 (0.75-5)	.26
Chest tube output, 0-24 h, mL	96 (69.7-134)	99.4 (77.6-140.7)	.74
Chest tube output, 24-48 h, mL	50.7 (24.0-72.0)	44.5 (24.5-58.7)	.73

Volumes in milliliters. *IQR*, Interquartile range; *OR*, operating room; *pRBC*, packed red blood cells; *FFP*, fresh-frozen plasma; *ICU*, intensive care unit; *Hgb*, hemoglobin; *Plt*, platelet; *Fib*, fibrinogen; *INR*, international normalized ratio.

**Table E5:** Unadjusted outcome variable results for whole blood and standard prime blood groups by Society of Thoracic Surgeons-European Association for Cardio-Thoracic Surgery 5 subgroup

STAT 5 outcome measures	Whole blood (n = 13)	Standard blood (n = 13)	*P* value
	Median (IQR)	Median (IQR)	
No. of OR donors	4 (3-5)	5 (4-8)	.04
OR pRBC, mL	137 (0-250)	250 (160-340)	.04
OR cryoprecipitate, mL	20 (0-40)	40 (25-48)	.04
OR FFP, mL	0 (0-0)	70 (50-100)	<.001
OR platelets, mL	55 (30-80)	100 (70-110)	<.001
Postoperative ICU Hgb, g/dL	14.9 (12.3-16.6)	13.8 (13.6-14.8)	.86
Postoperative ICU Plt, ×10^3^/μL	162 (125-225)	177 (141-293)	.44
Postoperative ICU Fib, mg/dL	214 (186-225)	267 (236-323)	.006
Postoperative ICU INR	1.4 (1.3-1.4)	1.4 (1.3-1.5)	.89
Sternal closure time, d	3 (0-8)	4 (4-5)	.72
Chest tube output, 0-24 h, mL	101.2 (80-119)	130 (98-140)	.10
Chest tube output, 24-48 h, mL	30.0 (20.0-57.1)	65.0 (45.0-70.0)	.04

Volumes in milliliters. *IQR*, Interquartile range; *OR*, operating room; *pRBC*, packed red blood cells; *FFP*, fresh-frozen plasma; *ICU*, intensive care unit; *Hgb*, hemoglobin; *Plt*, platelet; *Fib*, fibrinogen; *INR*, international normalized ratio.
